# Exposing Selection and Genetic Linkage in the Evolutionary Enigmatic Balanced Lethal System in *Triturus* Newts

**DOI:** 10.1002/ece3.71591

**Published:** 2025-06-12

**Authors:** Willem R. M. Meilink, Milena Cvijanović, Manon C. de Visser, James France, Ana Ivanović, Anagnostis Theodoropoulos, Tijana Vučić, Ben Wielstra

**Affiliations:** ^1^ Naturalis Biodiversity Center Leiden the Netherlands; ^2^ Institute of Biology Leiden Leiden University Leiden the Netherlands; ^3^ Department of Evolutionary Biology, Institute for Biological Research “Siniša Stanković”, National Institute of Republic of Serbia University of Belgrade Belgrade Serbia; ^4^ Blijdorp Conservation & Science Center Royal Rotterdam Zoological & Botanical Gardens Rotterdam the Netherlands; ^5^ Faculty of Biology University of Belgrade Belgrade Serbia

**Keywords:** chromosome 1 syndrome, crossing over, KASP genotyping, recombination, supergene

## Abstract

A balanced lethal system is a genetic disease that results in the loss of half of the reproductive output. The best‐known balanced lethal system is found in newts of the genus *Triturus.* In these newts, two different versions of chromosome 1, named *1A* and *1B*, do not recombine along a particular region. Only individuals with both versions are viable, whereas those that possess the same version twice die. We cross two *Triturus* species to generate F1 and F2 hybrid offspring. This allows us to follow 30 species‐specific single nucleotide polymorphisms (SNPs) from across the genome, including the non‐recombining region involved in the balanced lethal system, over three generations. We confirm that individuals carrying the same chromosome version twice die, whereas those that possess both versions survive. Recombination is observed outside, but not within, the region associated with the balanced lethal system. Our experiment provides a clear‐cut example of Mendelian inheritance with a deadly twist, well suited to teach basic principles of natural selection and genetic linkage.

## Graphical abstract

A balanced lethal system is a genetic disease that results in the loss of half of the reproductive output. Recombination is observed outside, but not within, the region associated with the balanced lethal system. Our experiment provides a clear‐cut example of Mendelian inheritance with a deadly twist, well suited to teach basic principles of natural selection and genetic linkage.

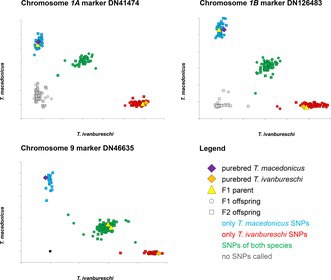

## 1 Introduction

Natural selection is supposed to maximize individual fitness (Darwin 1859). In this context, balanced lethal systems represent an evolutionary enigma, because they kill off exactly half of the offspring during embryonic development (Muller 1918; Wielstra 2020). They work as follows: in an affected, diploid organism, there are two versions of a particular chromosome, let us call them *A* and *B*. There is no crossing over between *A* and *B*; rather they are each inherited as a single unit, as a “supergene” (Schwander et al. 2014; Berdan, Flatt, et al. 2022). The lack of crossing over that defines supergenes is often caused by chromosome rearrangements (Thompson and Jiggins 2014; Gutiérrez‐Valencia et al. 2021). Because both versions *A* and *B* are required for survival, all adults in the population by definition possess one copy of version *A* and one copy of version *B*. Adults randomly transmit either version *A* or version *B* in each of their haploid egg or sperm cells. Upon fertilization, two gametes fuse to form a diploid zygote. Four combinations are possible, each with a 25% chance, following the rules of Mendelian inheritance: *AA*, *AB*, *BA*, or *BB*. Only two of these options, the *AB* and *BA* genotypes, are viable. The other half, the *AA* and *BB* genotypes, perish before reproducing (Muller 1918; Wielstra 2020).

The most famous balanced lethal system is named “chromosome 1 syndrome” (Macgregor 1991). For this balanced lethal system, both the evolutionary clade affected (the monophyletic genus *Triturus*, comprising the crested and marbled newts) and the genomic region responsible (the long arm of chromosome 1), are clearly delineated. From karyotyping it is known that all adult *Triturus* newts consistently carry two versions of chromosome 1, called *1A* and *1B* (Macgregor and Horner 1980; Sims et al. 1984; Sessions et al. 1988). Although chiasmata can form along the rest of chromosome 1, there is no crossing over between the long arms of *1A* and *1B* (Callan and Lloyd 1960; Mancino and Nardi 1971). The region covered by these supergenes occupies a significant amount of physical space on chromosome 1 (Macgregor 1991). Version *1A* lacks crucial genes that are only present on version *1B*, and the other way around (de Visser, France, Paulouskaya, et al. 2024; France et al. 2024). Consequently, the 50% of offspring that possess either two copies of *1A* or two copies of *1B* lack particular crucial genes and die during the tailbud phase, approximately halfway through embryological development (Sessions et al. 1988; Vučić et al. 2024). Thus, in *Triturus* embryos there are multiple genotypes that differ in the number of copies of *1A* and *1B*: two types of heterozygotes, *1A1B* = *1B1A*, and two types of homozygotes, *1A1A* and *1B1B*.

**FIGURE 1:**
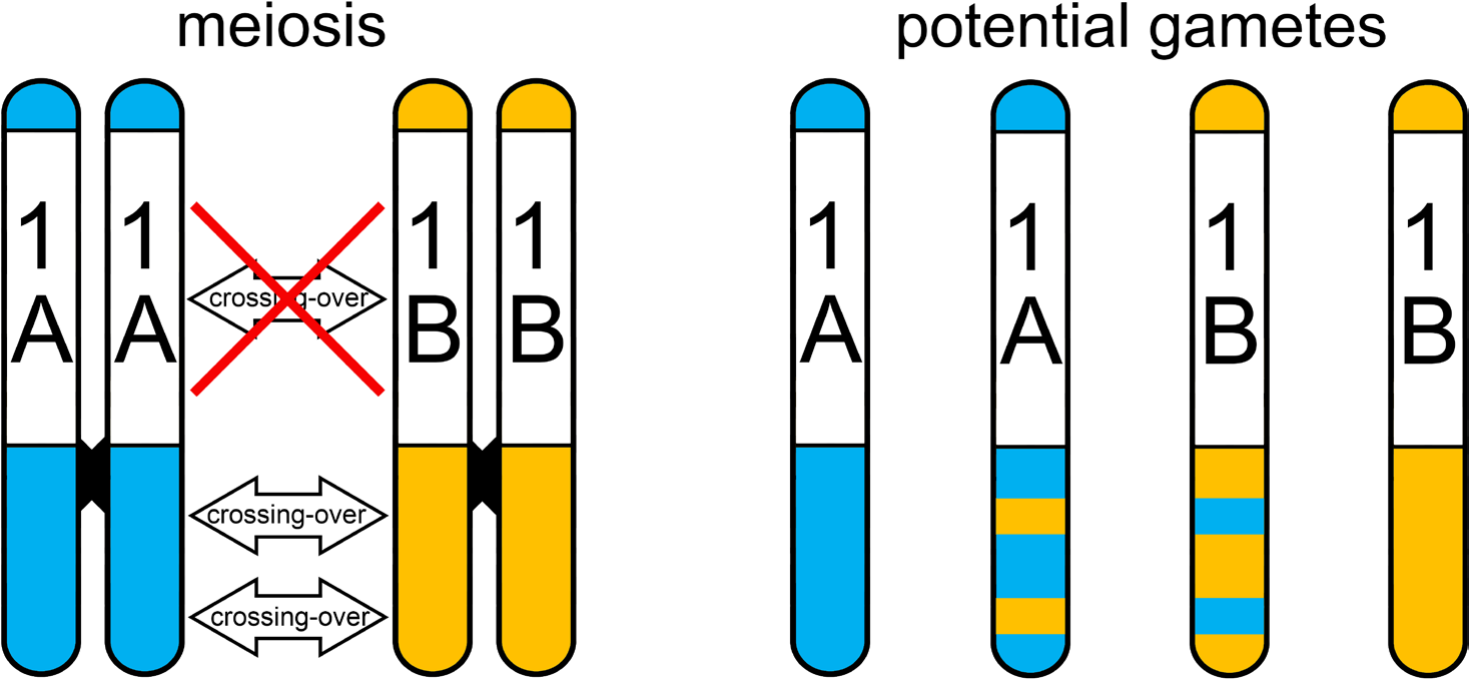
The role of suppressed recombination in the balanced lethal system in *Triturus* newts. During meiosis, chromatids may exchange genetic material where they form chiasmata (arrows left). However, chromosome 1 in *Triturus* does not form chiasmata between the unique *1A* and *1B* genomic regions. Therefore, gametes may contain recombined regions outside, but not within, the *1A* and *1B* regions.

If two species have species‐diagnostic and individually recognizable alleles for *1A* or *1B*‐linked markers, the number of *1A* and *1B* copies present (0, 1, or 2) can be genotyped in their F1 hybrids—they can be counted. To this aim, we use Kompetitive Allele Specific PCR (KASP), a cheap and fast technique to genotype species‐specific single nucleotide polymorphisms (SNPs) for a large number of individuals (Semagn et al. 2014). For regular diploid markers, two distinct alleles should always be present. Normally, when F1s are crossed with each other to produce F2s, recombination shuffles the genes of the homologous chromosomes derived from the two parental species. However, this would not be the case for *1A* or *1B*. Since they are shielded from crossing over, *1A* and *1B* would be expected to be transmitted to the F2 generation as a single unit—as a supergene (Figure 1). Here, we leverage interspecific hybrids with two aims: (1) to count the number of copies for chromosome *1A* and *1B*, and (2) to determine whether recombination is prevented between chromosome *1A* and *1B*. We test the following hypotheses:

- F1 hybrid embryos collected before developmental arrest occurs should have all chromosome 1 genotypes represented (*1A1A*, *1A1B*, *1B1A*, and *1B1B*);
- F1 hybrid embryos that have experienced arrested development should have two copies of either chromosome *1A* or *1B*;
- F1 hybrid embryos that have survived the tailbud phase, in which developmental arrest occurs, should have a single copy of chromosome *1A* and a single copy of chromosome *1B*;
- F2 hybrid embryos show recombination across the genome, except for between the affected regions of *1A* and *1B*.

## 2 Methods

### 2.1 Samples

**FIGURE 2:**
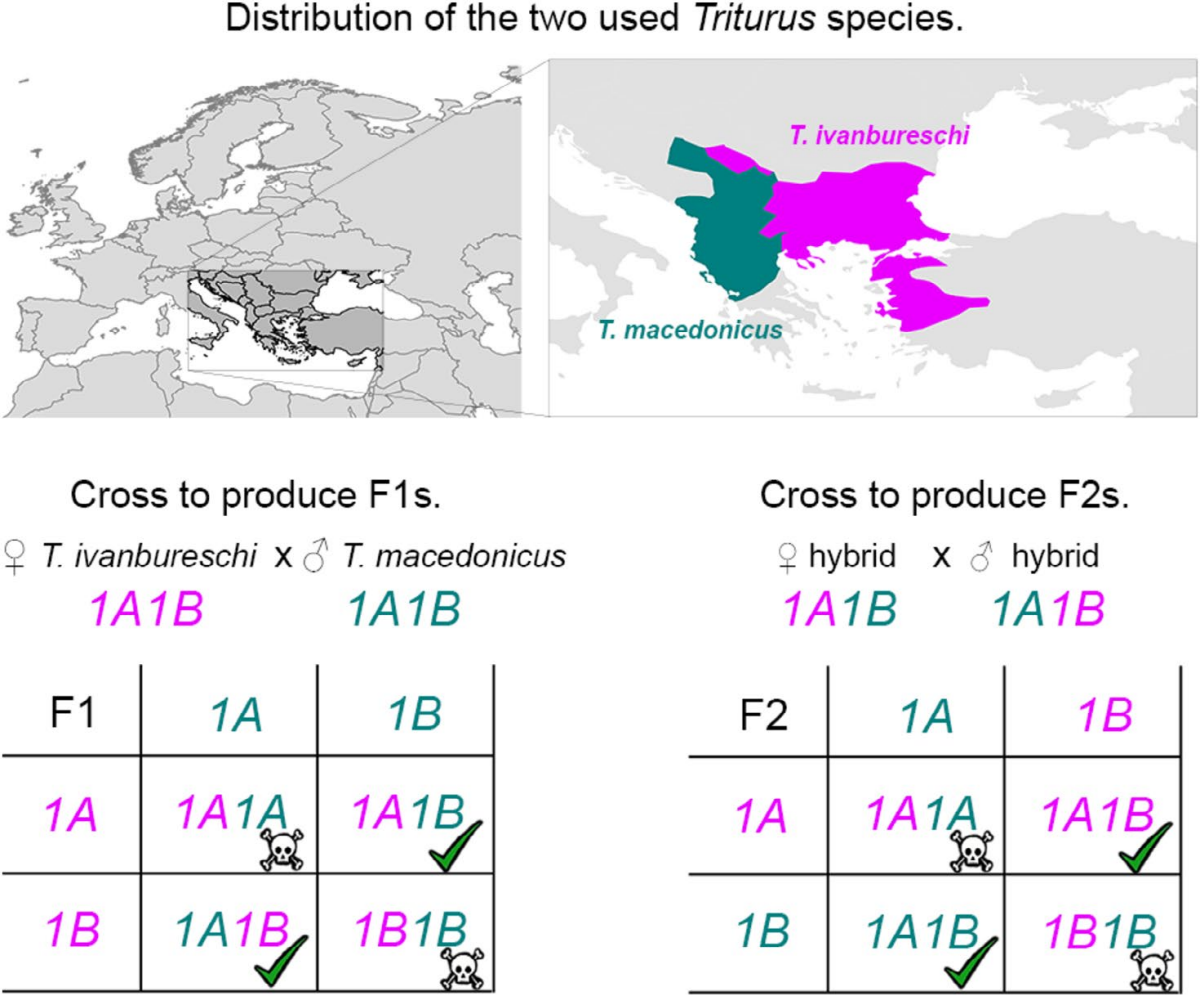
The distribution of the two *Triturus* species and the their interspecific crosses used in this study. *Triturus ivanbureschi* (magenta) and *T. macedonicus* (teal) are distributed in south‐eastern Europe and Turkey and meet at a hybrid zone on the Balkan Peninsula. F1 hybrids derive from a cross between a purebred female *T. ivanbureschi* and a purebred male *T. macedonicus* . Therefore, viable offspring should possess chromosome *1A* from one parent and chromosome *1B* from the other. F2 hybrids derive from a cross between two F1 hybrids with an opposite chromosome 1 constitution. Therefore, viable offspring should possess chromosomes *1A* and *1B* from either one or the other species.

We use hybrids between the Balkan crested newt ( *Triturus ivanbureschi* ) and the Macedonian crested newt ( *T. macedonicus* ). These two species are distributed in southeastern Europe and Turkey, and they hybridize in the wild where their ranges meet (Figure 2; Wielstra et al. 2017). A breeding colony of both species has been established at the Institute for Biological Research “Siniša Stanković,” University of Belgrade, Belgrade, Serbia (Vučić et al. 2024). We created F1 hybrids by crossing a female *T. ivanbureschi* with a male *T. macedonicus* (Figure 2). Next, two F1 hybrids were crossed to produce F2 hybrids; the male had a chromosome *1A* from *T. macedonicus* and a chromosome *1B* from *T. ivanbureschi*, and the female had the opposite constitution; this strategy ensures that the number of *1A* and *1B* copies present could be counted and putative crossing over events could in principle be established (Figure 2). In total, we produced five classes of embryos:

1. 48 F1 hybrids collected before the embryonic stage in which developmental arrest occurs;
2. 24 F1 hybrids that experienced developmental arrest;
3. 24 F1 hybrids that survived the balanced lethal system;
4. 48 F2 hybrids that experienced developmental arrest;
5. 48 F2 hybrids that survived the balanced lethal system.

Thus, combined, 196 samples were investigated in total (192 embryos, the two purebred parents of the F1s and the two F1 parents of the F2s).

### 2.2 SNP Design

We first obtained sequence data from c. 7 k nuDNA markers using “NewtCap,” a target enrichment by sequence capture protocol (Wielstra et al. 2019; de Visser, France, McCartney‐Melstad, et al. 2024), for nine individuals (three individuals each, from three populations distributed throughout the range) for both *T. ivanbureschi* and *T. macedonicus* (Table S1; DNA extractions taken from Wielstra et al. 2017). We retreived a mean of 5.72 million read pairs per sample (min: 4.23 million, max: 7.36 million). Data was processed via the bioinformatics pipeline described for “NewtCap” (de Visser, France, McCartney‐Melstad, et al. 2024). The raw VCF files were filtered to only include biallecic single nucleotide polymorphisms (SNPs) with a mapping quality greater than 10 with VCFtools version 0.1.16 (Danecek et al. 2011) before the *‐consensus* function of BCFtools version 1.18 (Danecek et al. 2021) was used to generate individual FASTA sequences for each sample.

**FIGURE 3:**
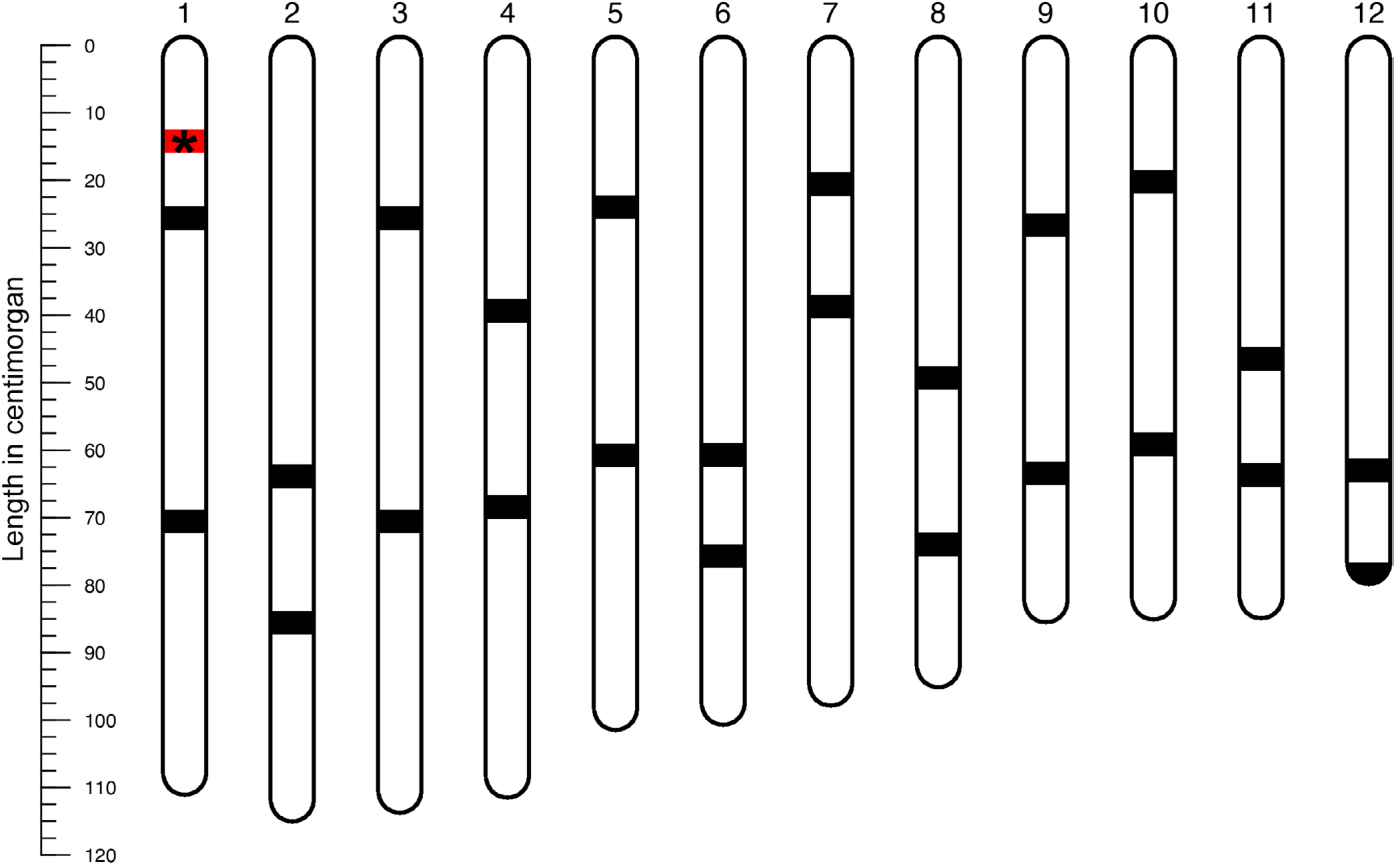
The location of the 30 SNP markers studied on the *Triturus* linkage map. Black bars represent the location of SNP markers on the 12 linkage groups comprising the *Triturus* linkage map, whereas the single red bar labeled with * represents the location of the three SNP markers on chromosome *1A* and *1B* (France et al. 2024).

For 4226 of the nuDNA markers, the position on the genome is known from linkage mapping (France et al. 2024) and dozens of markers private to either *1A* or *1B* have been identified (de Visser, France, Paulouskaya, et al. 2024; France et al. 2024). For each of the two species, we imported and aligned the nine available sequences for potential markers and built consensus sequences using UGENE version 46.0 (Okonechnikov et al. 2012). Any site that showed an intraspecific SNP was denoted using the appropriate IUPAC code. We then aligned the consensus sequences of both species to identify potential species‐specific SNPs. Given the suggested origin of the balanced lethal system in *Triturus* by an unequal exchange of genetic material (Sessions et al. 1988; France et al. 2024), *1A* and *1B* markers may be present in two copies on one particular chromosome. Therefore, we visually compared mapped sequence reads in IGV version 2.12.3 (Robinson et al. 2011; Thorvaldsdóttir et al. 2012) to ensure that SNPs on *1A* and *1B* markers were unambiguously species‐diagnostic. We selected 30 species‐diagnostic SNPs for *T. ivanbureschi* versus *T. macedonicus* that are distributed across the genome (Figure 3; Table S2). These include three SNP markers on *1A*, three SNP markers on *1B*, two SNP markers on the recombining part of chromosome 1, and two SNP markers on each of the remaining chromosomes 2–12.

### 2.3 KASP Genotyping

We genotyped our 30 SNP markers using Kompetitive Allele‐Specific PCR (KASP) to determine if *T. ivanbureschi* and/or *T. macedonicus* alleles are present. KASP involves fluorescence‐based genotyping (Semagn et al. 2014). The SNP variant present in each individual (both variants in the case of a heterozygote) is determined in uniplex assays, based on a common reverse primer and two allele‐specific forward primers—with a final base complementary to one of the two potential SNP variants—that also possess unique tail sequences. Two distinctly fluorescently labeled sequences present in the KASP master mix are complementary to each tail sequence. The labels are originally quenched and are only activated when the labeled sequences are incorporated during subsequent PCR cycles, with further cycling causing signal intensity to increase. Depending on which signal is emitted, it can be determined which SNP variant is present (none, one or the other, or both). Primers for the 30 SNP markers were designed in Kraken 23.11.7 (Table S2).

### 2.4 Multiplex PCR Genotyping

All samples were also independently genotyped for their chromosome 1 constitution (i.e., to determine if they were *1A1A*, *1B1B*, or *1A1B*/*1B1A*; this approach does not distinguish between the *1A* and *1B* of different *Triturus* species) with a multiplex PCR approach that includes both a *1A*‐ and a *1B*‐linked marker (Meilink et al. 2024) to confirm KASP genotyping results. The mxPCR was performed using QIAgen multiplex PCR master mix. PCR was performed in 12 μL reactions, containing 0.06 μL of all forward and reverse primers (at 10 μM concentration, resulting in a 0.05 μM end concentration of each primer), 6 μL QIAGEN multiplex PCR master mix, 4.64 μL purified water, and 1 μL of DNA. PCR conditions were as follows: a hot start for 15 min at 95°C, followed by 35 cycles of denaturation for 30 s at 95°C, annealing for 1 min at 56°C, extension for 1 min at 72°C, and a final 10‐min extension at 72°C.

## 3 Results

**FIGURE 4:**
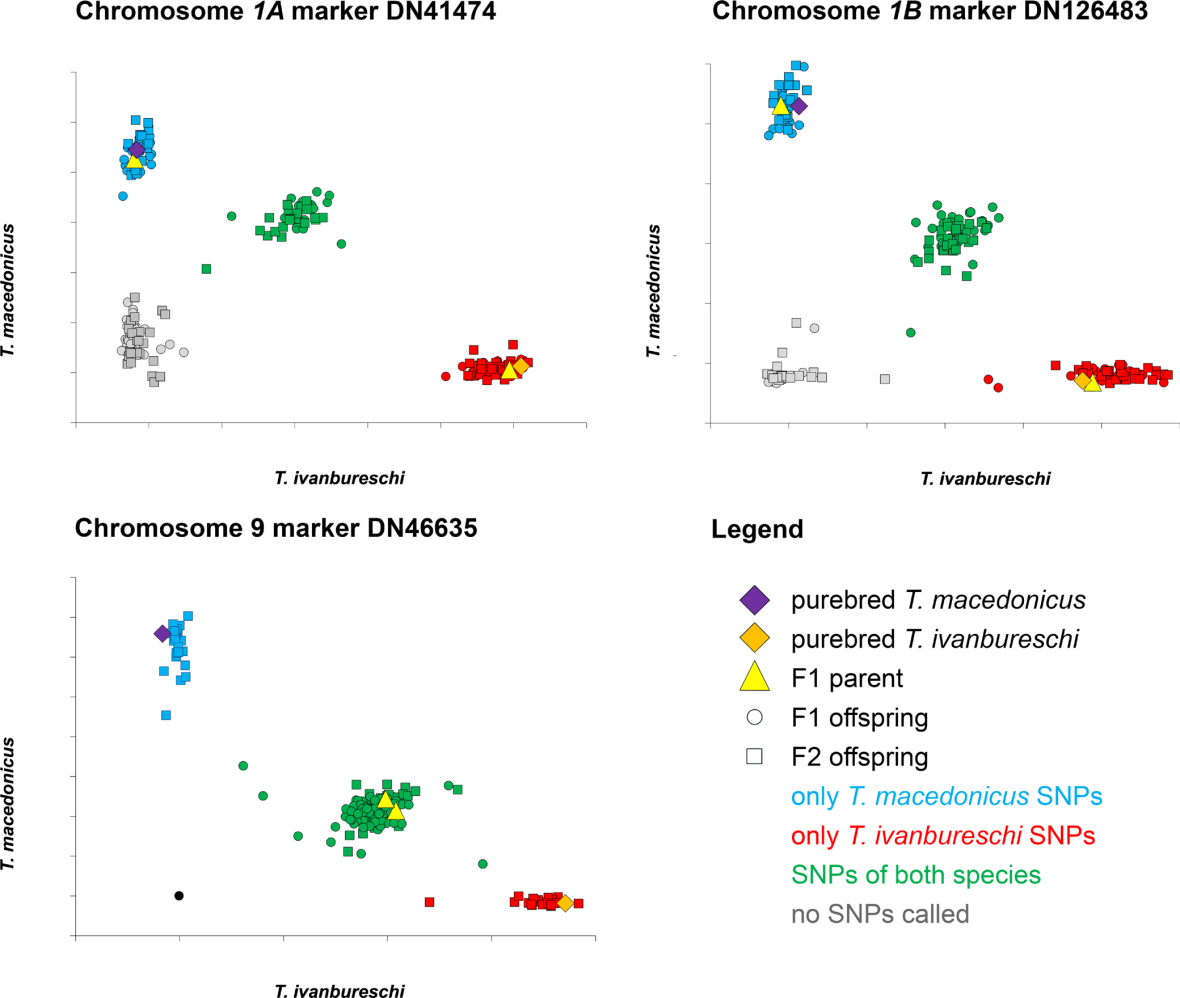
Example of KASP results for markers positioned on chromosomes *1A*, chromosome *1B* and chromosome 9. Based on fluorescence intensity (along the X‐ and Y‐axis), SNPs are called as either *T. macedonicus* (blue), or *T. ivanbureschi* (red), or both (green). Gray denotes that no SNP call was made, which could constitute a failed call or signal the absence of a particular marker. Purebred and F1 parents are highlighted in a different color and symbol.

The KASP results are highly accurate; examples of a *1A*, a *1B* and a “normal” SNP marker are shown in Figure 4 and the total dataset is available as Table S3. If we look at the 24 SNP markers not positioned on chromosome *1A* or *1B* across all 196 individuals, we observe 93/4704 (2.0%) failed calls (Table S3). All 96 F1 embryos are heterozygous for the 24 SNP markers that are not positioned on chromosome *1A* or *1B*, except for 6/4704 (0.3%) “wrong” homozygous calls (Figure 3; Table S3). Based on the 24 SNP markers not positioned on chromosome *1A* or *1B*, all F2 embryos show evidence of recombination and crossing over (Table S3).

The KASP‐based genotypes of individuals fully correspond to those based on multiplex PCR: in *1A1A* individuals, *1B* SNP markers are not called (and reflect an actual absence), and vice versa. Information for the three individual SNP markers for *1A* and the three individual SNP markers for *1B* is congruent across individuals. We see one (0.5%) exception: individual BW_0242 has zero copies of chromosome *1B* and two of *1A* (Table S3). For only one of the three *1A* SNP markers do we find the expected constitution of one *T. ivanbureschi* and one *T. macedonicus* chromosome *1A*. The second *1A* SNP marker is considered failed and the third appears to suggest two *T. ivanbureschi* chromosomes 1A are present, but also do not cluster within the *T. ivanbureschi* “cloud” of genotype calls. We consider this one outlier call artifactual.

The F1 hybrids in the embryo class collected before developmental arrest represent all genotypes: 12 *1A1A* (25.0%), 10 *1A1B* (20.8%; with *T. ivanbureschi 1A* and *T. macedonicus 1B*), 9 *1B1A* = (18.8%; with *T. macedonicus 1A* and *T. ivanbureschi 1B*) and 17 *1B1B* (35.4%). All embryos that experienced developmental arrest possess either two chromosomes *1A* or *1B* (4 *1A1A* vs. 20 *1B1B* in the F1s and 20 *1A1A* vs. 26 *1B1B* in the F2s). All embryos that survived the critical developmental stage possess both a chromosome *1A* and *1B* (13 with a *T. ivanbureschi 1A* and 11 with a *T. macedonicus 1A* in the F1s and 30 with a *T. ivanbureschi 1A* and 18 with a *T. macedonicus 1A* in the F2s). We see no evidence of crossing over between the *1A* and *1B* regions of chromosome 1 in F2 embryos.

## 4 Discussion

Balanced lethal systems represent an evolutionary paradox: they are incredibly maladaptive, yet are found in a wide variety of taxa (Wielstra 2020). They are assumed to represent a supergene with two versions: (1) that each possess unique lethal alleles, meaning that both versions are required for survival, and (2) that do not undergo crossing over, meaning the lethal alleles cannot be replaced by functional counterparts. We leverage interspecific hybrids and designed a KASP approach to genotype genome‐wide, species‐specific SNP markers to test hypotheses on selection and linkage in the balanced lethal system found in *Triturus* newts. Our experiment clearly illustrates how this balanced lethal system is inherited and maintained.

In F1 hybrid individuals, before the critical moment during embryonic development when 50% of offspring dies, all four possible genotypes are still present: individuals may show SNPs that are positioned on *1A*, on *1B*, or both (Figure 4). After the critical moment during embryonic development, only those individuals that show SNPs that are positioned on both *1A* and *1B* survive. Those individuals that only show SNPs that are positioned on either *1A* or *1B* die from the balanced lethal system. Our findings thus support predictions on which chromosome 1 constitution results in viable offspring (i.e., possessing both *1A* and *1B*) and which chromosome 1 constitution results in death in the balanced lethal system (i.e., possessing *1A* or *1B* twice) (Macgregor and Horner 1980; Horner and Macgregor 1985).

F2 hybrid individuals that experienced developmental arrests consistently have SNPs that are positioned on *1A* or *1B* from both parental species (Figure 2; Table S3). Those individuals that survived the balanced lethal system consistently have SNPs that are positioned on *1A* and *1B* from only one or the other parental species. Our findings thus support the prediction that crossing over occurs between SNPs positioned across the *Triturus* genome, except between those SNPs that are positioned on *1A* or *1B* (Callan and Lloyd 1960; Macgregor and Andrews 1977; Figure 1). This local lack of crossing over is in line with an absolute linkage between *1A* markers and an absolute linkage between *1B* markers.

The *Triturus* balanced lethal system exemplifies the linked inheritance of supergenes within a genomic background that experiences regular recombination. This Mendelian inheritance with a deadly twist observed in *Triturus* is well suited for teaching the basic principles of natural selection and genetic linkage (Meilink et al. 2021). The approach we take in the present study could also be applied to other balanced lethal systems reported in nature, in insects (Muller 1917, 1918; Dawson 1967) and plants (Steiner 1956; Golczyk 2011; Golczyk et al. 2014).

It seems counterintuitive that something so wasteful as a balanced lethal system would ever evolve in the face of natural selection. The huge mortality rate that recurs every generation appears to defy the basic tenets of evolutionary theory. Perhaps the individuals that survive the balanced lethal systems have a fitness advantage that outweighs the loss of half of the eggs? Although this would help explain the origin of a balanced lethal system, no evidence has been put forward yet to support this hypothesis. Actually, several studies involving evolutionary modeling have shown that, albeit under specific conditions, a balanced lethal system can become fixed in the population despite the associated negative fitness costs (Berdan, Blanckaert, et al. 2022; France et al. 2024). We hope that future studies can solve this evolutionary mystery.

## Author Contributions

**Willem R. M. Meilink:** conceptualization (equal), data curation (equal), investigation (equal), methodology (equal), writing – original draft (equal). **Milena Cvijanović:** methodology (supporting). **Manon C. de Visser:** methodology (supporting). **James France:** methodology (supporting). **Ana Ivanović:** methodology (supporting). **Anagnostis Theodoropoulos:** methodology (equal). **Tijana Vučić:** methodology (supporting). **Ben Wielstra:** conceptualization (equal), supervision (lead), writing – original draft (equal).

## Disclosure

Benefit Sharing: All generated data has been published in this paper and can be found in the Supporting Information.

## Conflicts of Interest

The authors declare no conflicts of interest.

## Supporting information


**Table S1.** Origin of the parental species used to obtain species‐diagnostic sequences.
**Table S2.** Marker, consensus sequences, and all primer sequences used. The # and * in the primer sequences indicate the location of the LGC‐patented dye‐complementary tail sequence.
**Table S3.** All samples and their respective classes (left three columns), genotypes, and markers/chromosome with SNP calls and fluorescence intensity.

## Data Availability

The Illumina sequencing reads generated for this study have been submitted to the NCBI Sequence Read Archive (SRA) and can be retrieved through BioProject PRJNA1223945. All scripts utilized can be found in the following Zenodo repository: https://zenodo.org/records/15608534.
